# Diagnostic Performance of Machine Learning Algorithms for Predicting Heart Failure in Diabetic Patients: A Systematic Review and Meta‐Analysis

**DOI:** 10.1002/edm2.70111

**Published:** 2025-09-18

**Authors:** Pooya Eini, Peyman Eini, Homa Serpoush, Mohammad Rezayee

**Affiliations:** ^1^ Cardiovascular Research Center Rajaie Cardiovascular Institute Tehran Iran; ^2^ Infectious Disease Research Center Hamadan University of Medical Sciences Hamadan Iran; ^3^ Hamadan University of Medical Sciences Hamadan Iran; ^4^ College of Human Medicine Michigan State University East Lansing Michigan USA

**Keywords:** diabetes, diagnostic accuracy, heart failure, machine learning, predictive modelling

## Abstract

**Background:**

Heart failure is a significant complication in diabetic patients, and machine learning algorithms offer potential for early prediction. This systematic review and meta‐analysis evaluated the diagnostic performance of ML models in predicting HF among diabetic patients.

**Methods:**

We searched PubMed, Web of Science, Embase, ProQuest, and Scopus, identifying 2830 articles. After deduplication and screening, 16 studies were included, with 7 providing data for meta‐analysis. Study quality was assessed using PROBAST+AI. A bivariate random‐effects model (Stata, midas, metadta) pooled sensitivity, specificity, likelihood ratios, and diagnostic odds ratio (DOR) for best‐performing algorithms, with subgroup analyses. Heterogeneity (*I* ^2^) and publication bias were assessed.

**Results:**

This meta‐analysis of seven studies evaluating machine learning models for heart failure detection demonstrated a pooled sensitivity of 84% (95% CI: 0.75–0.90), specificity of 86% (95% CI: 0.56–0.97), and an area under the ROC curve of 0.90 (95% CI: 0.87–0.93). The pooled positive likelihood ratio was 6.6 (95% CI: 1.2–35.9), and the negative likelihood ratio was 0.17 (95% CI: 0.08–0.36), with a diagnostic odds ratio of 39 (95% CI: 4–423). Significant heterogeneity was observed, primarily related to differences in study populations, machine learning algorithms, dataset sizes, and validation methods. No significant publication bias was detected.

**Conclusion:**

Machine learning models demonstrate promising diagnostic accuracy for heart failure detection and have the potential to support early diagnosis and risk assessment in clinical practice. However, considerable heterogeneity across studies and limited external validation highlight the need for standardised development, prospective validation, and improved interpretability of ML models to ensure their effective integration into healthcare systems.

## Graphical abstract

Machine learning models demonstrate high diagnostic accuracy for predicting heart failure in patients with type 2 diabetes, outperforming traditional risk models. Their ability to integrate diverse clinical data highlights their potential for early detection and risk stratification in clinical practice.

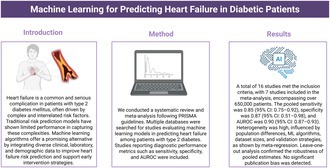

## 1 Introduction

Heart failure (HF) is a serious cardiovascular complication in patients with type 2 diabetes mellitus (T2DM), often driven by poor glycemic control, coronary artery disease, and ageing [1]. In diabetic populations, markers like HbA1c and fasting glucose are important predictors of HF [2]. They reflect underlying mechanisms such as insulin resistance and diabetic cardiomyopathy [3]. Among patients with T2DM, about 3.5% are hospitalised for HF over 360,258 person‐years, corresponding to 5.2 events per 1000 person‐years [4]. The cumulative 5‐year event rate reaches 2.0%, highlighting the urgent need for early risk identification [3]. Risk scores that include factors like systolic blood pressure, BMI, and eGFR have shown good discrimination for predicting HF at 5 and 10 years [5]. High‐risk groups may face up to a 15‐fold increase in HF risk compared to individuals with normal glucose levels [2]. Yet, despite reasonable calibration, these traditional models perform modestly in prediabetic populations and often fail to account for dynamic risk factors such as fluctuations in glycemic control or medication use [6].

Machine learning (ML) algorithms offer a promising alternative, leveraging advanced pattern recognition to address the limitations of traditional statistical approaches [7]. Unlike basic risk scores, ML models can integrate high‐dimensional data such as HbA1c variability, renal biomarkers, and prior cardiovascular events to enhance predictive accuracy [8]. Given the wide variation in HF risk across age groups and risk categories, there is a clear need for better tools to support early detection and risk stratification in diabetes care.

This systematic review and meta‐analysis holds significant importance in advancing the field of HF prediction in diabetic patients by providing the comprehensive synthesis of ML‐based diagnostic performance. While individual studies have demonstrated ML's potential, the lack of a unified evaluation has hindered clinical adoption. By pooling data across diverse studies, our work quantifies ML's diagnostic accuracy and compares it to traditional methods, addressing gaps in understanding heterogeneity and generalisability.

## 2 Method

### 2.1 Search Strategy

The study protocol is registered at osf.io/5bcfd/ (Open Science Framework) and we adhered to Preferred Reporting Items for Systematic Review and Meta‐analyses Protocols (PRISMA) [9]. We systematically searched five electronic databases—PubMed, Web of Science (WoS), Embase, ProQuest, and Scopus—for relevant studies published up to June 13, 2025. The search strategy combined terms related to machine learning, heart failure, and diagnostic accuracy, with no language restrictions applied. A total of 2830 articles were identified and after removing duplicates, 1527 unique articles were screened. Full search syntax for different databases is available in Table S1.

### 2.2 Study Selection

**FIGURE 1:**
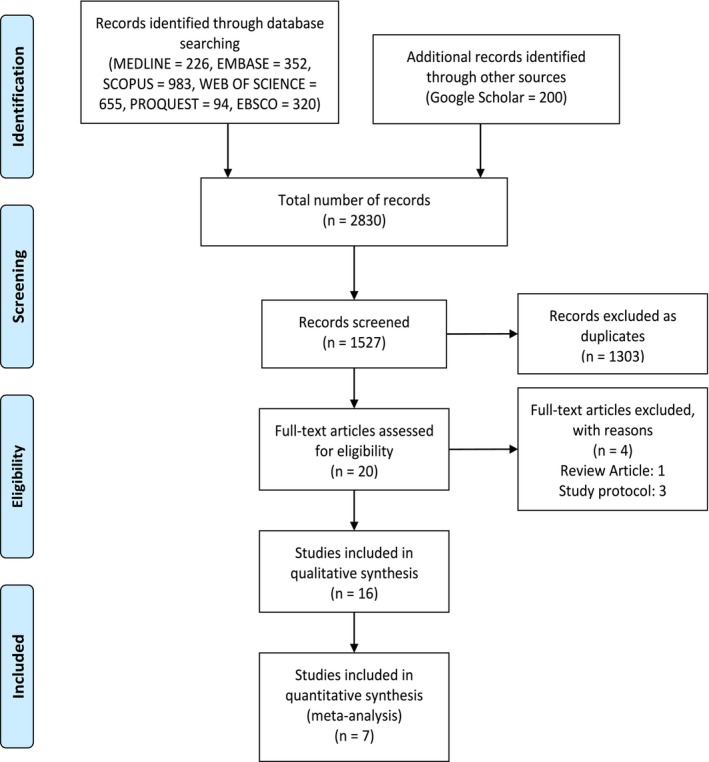
PRISMA flowchart for study screening and selection.

The screening process involved two stages. First, titles and abstracts of 1527 articles were independently reviewed by two investigators to assess eligibility based on predefined inclusion criteria: studies reporting diagnostic performance metrics (e.g., sensitivity, specificity, accuracy) of machine learning algorithms for HF prediction in adults using a test set with defined cases (HF) and controls (no HF). Discrepancies were resolved by consensus or consultation with a third reviewer. This initial screening yielded 20 articles selected for full‐text review. In the second stage, full texts were evaluated for data availability, methodological quality, and relevance to the meta‐analysis objectives. Studies lacking sufficient data (e.g., contingency tables or derivable metrics) or not meeting quality standards were excluded. Ultimately, 16 articles were included in the study, of which 7 provided adequate data for quantitative synthesis in the meta‐analysis (Figure 1).

### 2.3 Data Extraction

Data extraction was performed independently by two reviewers, capturing study characteristics (algorithm type, sample size, HF prevalence), performance metrics (sensitivity, specificity, accuracy), and test set details (cases and controls).

### 2.4 Quality Assessment

Quality assessment of the included studies was conducted using the PROBAST + AI tool, tailored for prediction model studies incorporating artificial intelligence [10]. This tool evaluated the risk of bias and applicability across domains such as participant selection, predictors, outcomes, and analysis, ensuring the robustness of the meta‐analysis. Studies were assessed independently by two reviewers, with disagreements resolved through discussion.

### 2.5 Statistical Analysis

Statistical analysis was performed using Stata version 18 with the midas and metadta packages. We employed a bivariate random‐effects model to pool sensitivity, specificity, positive likelihood ratio (LR+), negative likelihood ratio (LR−), and diagnostic odds ratio (DOR) across studies, accounting for the correlation between sensitivity and specificity. The summary receiver operating characteristic (SROC) curve and area under the curve (AUROC) were estimated to assess overall diagnostic accuracy. Heterogeneity was evaluated using the *I* ^2^ statistic and *χ* ^2^ test (Q statistic), with *I* ^2^ values interpreted as low (< 25%), moderate (25%–50%), or substantial (> 50%). Publication bias was assessed using a regression‐based test for small‐study effects (Deeks' method). For meta‐regression analysis, a random‐effects meta‐regression was conducted to explore the impact of machine learning model type on diagnostic performance. The Restricted Maximum Likelihood (REML) method was employed, with within‐study standard errors specified for the log DOR estimates.

### 2.6 Generative AI

In the preparation of this article, the authors utilised the Grammarly application to enhance linguistic accuracy and clarity. The manuscript underwent meticulous double‐checking to ensure precision, and the authors assume full responsibility for the integrity and originality of the content presented herein.

## 3 Result

### 3.1 Study Characteristics

This systematic review included 16 studies published between 2019 and 2025. The majority were observational studies (75%), with the remainder comprising clinical trials (12.5%), cohort studies (6.25%), and cross‐sectional studies (6.25%). The studies were conducted across a diverse range of countries, including the USA, Canada, Italy, Japan, South Korea, Australia, Uzbekistan, Poland, Spain, Hong Kong, and Sweden.

Validation methods varied among studies. Most used internal validation, with 81.25% employing k‐fold cross‐validation (predominantly 5‐ or 10‐fold), while a smaller proportion (18.75%) used split‐sample validation or external cohorts. Only a few studies incorporated robust external validation, which may influence the generalisability of the results.

The included models were trained and validated on a combined total of 845,816 participants in training sets, 237,747 in validation sets, and 122,118 in test sets. The dataset sizes ranged from fewer than 100 participants to large‐scale populations exceeding 600,000 individuals. Approximately 31% of studies analysed datasets larger than 100,000 participants, whereas 38% used datasets with fewer than 10,000 participants.

Predictors most commonly included demographics (age, sex), clinical variables (comorbidities, vital signs), laboratory data (biomarkers, electrolytes, blood indices), medication histories, and diagnostic codes. Several studies also used advanced predictors, such as composite risk indices, electrocardiographic parameters, and disease‐specific biomarkers, reflecting a broad spectrum of clinical data inputs.

The median follow‐up duration across studies was 47.4 months, though 37.5% of studies did not report follow‐up times explicitly. This variation in follow‐up likely contributed to differences in outcome definitions and model performance across studies.

Patient characteristics were inconsistently reported. However, the calculated mean age of participants was 50.8 years, and the average proportion of male participants was 53.1%, which aligns with typical heart failure demographics but highlights variability among study populations.

A wide variety of machine learning algorithms were tested, with each study evaluating multiple models. The most frequently selected best‐performing algorithms were Random Forest (18.8%) and Gradient Boosting (XGBoost) (18.8%), followed by Random Survival Forest (12.5%) and other models including Neural Networks, Deep Learning with GRU, Proportional Hazards Neural Networks, Stacked Models, Multinomial Logistic Regression, Explainable Boosting Machine, Light Gradient Boosting Machine, and Stochastic Gradient Boosting (each selected in 6.2% of studies). These results reflect the diverse algorithmic landscape in ML‐based heart failure prediction research. Detailed study characteristics are available in Table 1.

**TABLE 1:** included studies characteristics.

First author/year	Type of study	Country	Validation	Input characteristics	Selected features	Number of patients in training/test/validation group	Follow‐up (months)	Mean age/male %	Machin learning algorithms	Best predictor algorithm
Segar et al. (2019) [6]	Clinical trial	USA—Canada	Internal validation used 50% development and 50% validation split with—external validation in ALLHAT cohort	Clinical features (demographics, clinical variables, laboratory data, electrocardiographic parameters, baseline antihyperglycemic therapies, treatment randomization)	10	4378/7378/10,819	ACCORD: Median 58.8 months (4.9 years) ALLHAT: Median 57.6 months (4.8 years)	61.7/61.5%	Random Survival Forest (RSF)	RSF
Angraal et al. (2020) [11]	RCT	United States, Canada, Argentina, Brazil	5‐fold cross‐validation	Haemoglobin level, blood urea nitrogen (BUN), KCCQ variables, time since previous HF hospitalisation, glomerular filtration rate, and blood glucose levels.	68	1237/353/177	36	Mean age not reported; 50.1% male	Logistic Regression (LR) with forward selection, LR with lasso regularisation, Random Forest (RF), Gradient Descent Boosting (XGBoost), Support Vector Machine (SVM)	Random Forest (RF)
Lee et al. (2021) [12]	Observational study	Hong Kong	5‐fold cross‐validation	HbA1c mean and variability, HDL‐C mean and SD, baseline NLR, hypoglycemic frequency, lipid parameters: total cholesterol, LDL‐C, HDL‐C, triglycerides	22	17,630/5037/2519	132	63/50.4%	Random Survival Forests	Random Survival Forests
Longato et al. (2021) [13]	Observational study	ITALY	5‐fold cross‐validation	Clinical features (demographics, clinical variables, laboratory data, electrocardiographic parameters)	22	150,273/42,935/21,468	60	69/55%	Deep Learning model with GRU	Deep Learning model with GRU
Kanda et al. (2022) [8]	Observational study	Japan	80% training, 20% internal validation split; external validation using a separate dataset	Clinical features (patient demographics, ATC diagnosis codes, laboratory values)	60	173,643/43,410/16,822	60	Not Reported	Random Forest (RF), Logistic Regression (LR), Gradient Boosting (XGBoost), Deep Learning (Multilayer Perceptron), Cox Proportional Hazards	XGBoost
Abegaz et al. (2023) [14]	Observational study	USA	5‐fold cross‐validation	Clinical features (demographics, electrolytes, blood indices, disease‐specific biomarkers, physical measurements)	30	7247/1812/Not Reported	Not Reported	age > 18/42.6%	Random Forest (RF), Logistic Regression (LR), Extreme Gradient Boosting (XGBoost), Weighted Ensemble Model (WEM)	XGBoost
Gandin et al. (2023) [15]	Cohort Study	Italy	10‐fold cross‐validation	Clinical features (medical information, diagnostic codes, laboratory tests, procedures, cardiovascular drug prescriptions, comorbidities)	20	7430/1592/1592	65	72/42%	Proportional Hazards Neural Network (PHNN)	PHNN
Wang et al. (2023) [16]	Observational study	USA	Bootstrap method used for internal validation	Clinical features (demographics, self‐reported medical history, medication status, smoking/alcohol consumption, physical measurements, laboratory data)	6	2469/1058/Not Reported	Not Reported	59.62/57.5%	Logistic Regression (LR), Random Forest (RF), Classification and Regression Tree (CART), Gradient Boosting Machine (GBM), Support Vector Machine (SVM)	Random Forest (RF)
Alimova et al. (2023) [17]	Observational study	Uzbekistan	5‐fold cross‐validation	Clinical features (demographics, medical histories, medication history, clinical parameters, blood tests)	19	91/26/13	24	Not Reported	Random Forest (RF), Logistic Regression (LR), Generalised Linear Models (GLM), Extra Trees, Neural Networks (NN)	Neural Networks (NN)
Mora et al. (2023) [18]	Observational study	Spain	5‐fold cross‐validation	Clinical features (demographic data, medical histories, vital signs)	9	427,013/122,004/61,002	36	69.56/54.33%	Logistic Regression (LR), Decision Tree (DT), Random Forest (RF), Extreme Gradient Boosting (XGB), Stacked Model (based on LR combining LR, DT, RF, XGB)	Stacked Model
Nabrdalik et al. (2023) [19]	Observational study	Poland	5‐fold cross‐validation	Clinical features (demographic data, medical histories, vital signs)	10	1400/400/200	Not Reported	58.85/52%	Multinomial Logistic Regression (MLR)	Multinomial Logistic Regression (MLR)
Sang et al. (2024) [20]	Observational study	South Korea	Bootstrapping with 10,000 iterations	Clinical features (demographics, medical histories, medication history, clinical parameters, blood tests)	15	12,809/Not Reported/2019	36	62.5 ± 12.1/51.0%	Random Forest (RF), XGBoost (XGB), LightGBM (LGM), AdaBoost (ADB), Logistic Regression (LR), Support Vector Machine (SVM) with linear kernel	Random Forest (RF)
Soh et al. (2024) [21]	Observational study	Australia	5‐fold cross‐validation	Clinical features (demographic data, medical histories, vital signs, laboratory test results)	22	192/55/28	Not Reported	Not Reported	Explainable Boosting Machine (EBM)	Explainable Boosting Machine (EBM)
Kwiendacz et al. (2025) [22]	Observational study	Poland	5‐fold cross‐validation	Clinical features (demographic data, medical histories, vital signs, laboratory test results, medications)	10	781/335/Not Reported	37.2	67/57%	Logistic Regression (LR), Random Forest (RF), Support Vector Classification (SVC), Light Gradient Boosting Machine (LGBM), eXtreme Gradient Boosting (XGBM)	Light Gradient Boosting Machine (LGBM)
Bai et al. (2025) [5]	Cross‐sectional Study	USA	5‐fold cross‐validation	Clinical features (novel composite indices derived from anthropometric data, laboratory tests, and patient history)	7	1011/434/Not Reported	Not Reported	62.62/48.24%	Random Forest (RF), Logistic Regression (LR), XGBoost, Support Vector Machine (SVM), k‐Nearest Neighbours (kNN), Gradient Boosting, AdaBoost, Neural Network, Naive Bayes	XGBoost
Wändell et al. (2025) [23]	Observational study	Sweden	10‐fold cross‐validation	Clinical features (demographics, electrolytes, blood indices, disease‐specific biomarkers)	25	38,212/10,918/5459	36	≥ 30 years/60.4%	Stochastic Gradient Boosting (SGB)	Stochastic Gradient Boosting (SGB)

### 3.2 Pooled Analysis of Best‐Performing Models

**FIGURE 2:**
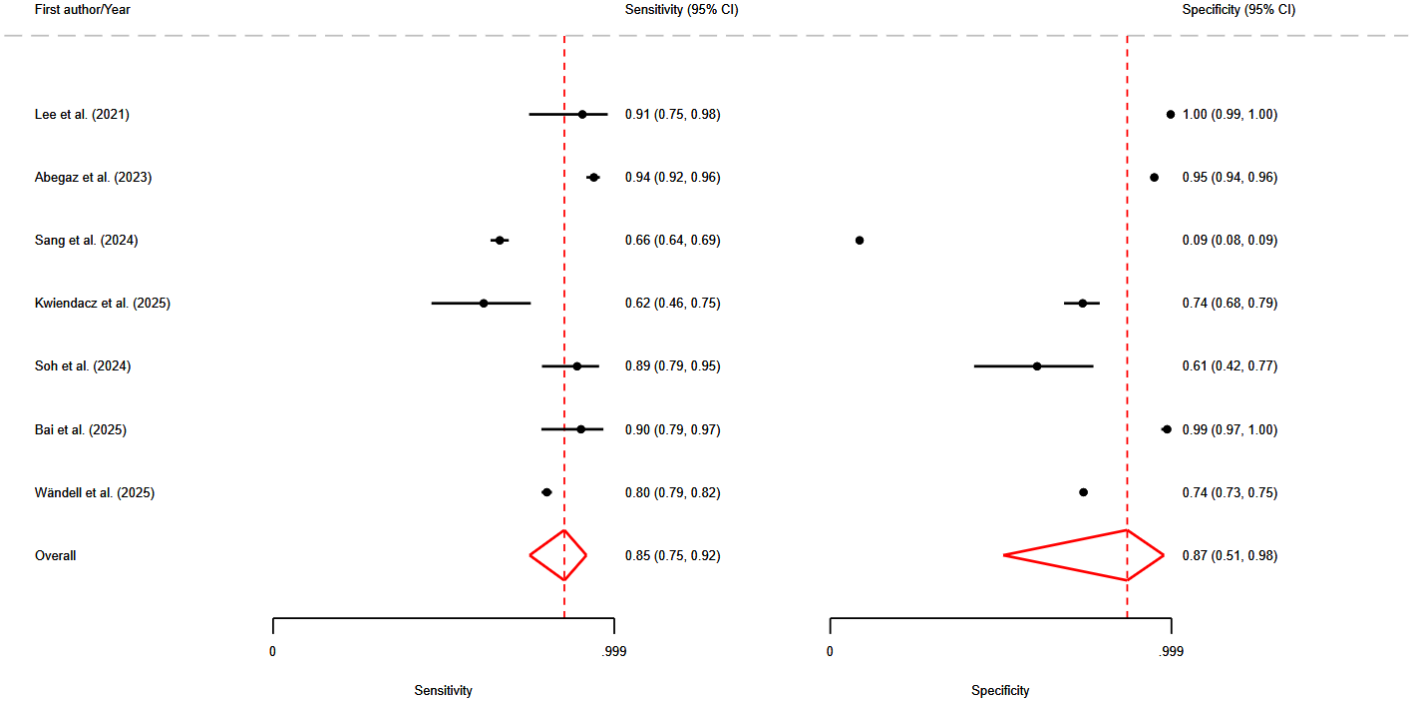
Forest plot for pooled sensitivity and specificity of Best‐Performing Models.

**FIGURE 3:**
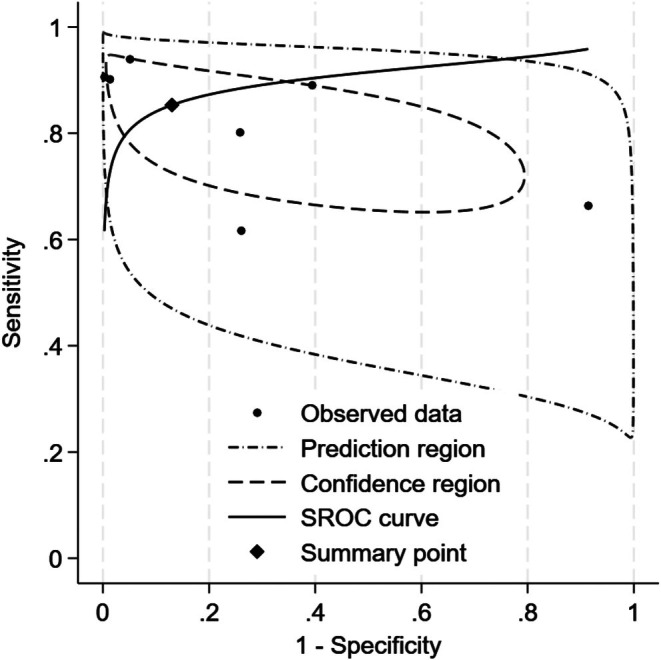
SROC curve of Best‐Performing Models.

In this analysis, we used the best‐performing machine learning model from each study, as reported by the original authors. Detailed performance metrics for different models are shown in Table 2. The pooled analysis demonstrated a sensitivity of 0.84 (95% CI: 0.75–0.90) and a specificity of 0.86 (95% CI: 0.56–0.97), based on a random‐effects model. These findings indicate a generally high level of diagnostic performance across the included studies. However, significant heterogeneity was observed, with an (*I* ^2^) of 83.17 for sensitivity and 87.77 for specificity, reflecting considerable differences in study outcomes (Figure 2). Additional pooled diagnostic performance metrics further underscored the strong potential of ML models in detecting heart failure. The pooled area under the receiver operating characteristic curve (AUROC) was 0.90 (95% CI: 0.87–0.93), suggesting excellent discriminatory power of these models in distinguishing patients with heart failure from those without the condition (Figure 3). The positive likelihood ratio (PLR) was 6.6 (95% CI: 1.2–35.9), indicating that individuals with heart failure were approximately 6.6 times more likely to have a positive test result from the ML models compared to individuals without heart failure. This level of PLR suggests that the models may have clinical utility in confirming a diagnosis of heart failure when test results are positive. The negative likelihood ratio (NLR) was 0.17 (95% CI: 0.08–0.36), a low value that implies a strong potential for ruling out heart failure when the ML test result is negative. Furthermore, the diagnostic odds ratio (DOR) was 39 (95% CI: 4–423), reflecting a high overall diagnostic effectiveness, although the wide confidence interval suggests variability across the studies. These findings demonstrate that ML models hold strong potential for clinical application in heart failure detection. The high sensitivity and low negative likelihood ratio support their use as screening tools, aiding in the early detection of heart failure and potentially reducing the risk of missed diagnoses. The high specificity and positive likelihood ratio suggest they may also be effective for confirming heart failure when used in conjunction with clinical assessment. The AUROC of 0.90 reflects excellent overall discriminative performance. Despite these promising results, the significant heterogeneity highlights the need for further standardisation and validation of ML models across diverse clinical populations and healthcare settings. Before widespread adoption into clinical practice, rigorous external validation and careful integration into diagnostic workflows will be essential to ensure reliable performance in real‐world scenarios.

**TABLE 2:** Performance metrics for different ML models. First row for each study indicates best performing algorithm.

First author/year	Predictor algorithm	Accuracy	Sensitivity (recall)	Specificity	Precision	F1 score	AUROC	AUROC (upper CI)	AUROC (lower CI)
Segar et al. (2019) [6]	RF	Not Reported	Not Reported	Not Reported	Not Reported	Not Reported	0.74	0.76	0.72
Angraal et al. (2020) [11]	RF	Not Reported	Not Reported	Not Reported	Not Reported	Not Reported	0.76	0.81	0.71
	LR with a forward selection	Not Reported	Not Reported	Not Reported	Not Reported	Not Reported	0.73	0.8	0.66
	LR with a lasso regularisation	Not Reported	Not Reported	Not Reported	Not Reported	Not Reported	0.73	0.79	0.67
	XGBoost	Not Reported	Not Reported	Not Reported	Not Reported	Not Reported	0.73	0.77	0.69
	SVM	Not Reported	Not Reported	Not Reported	Not Reported	Not Reported	0.72	0.81	0.63
Lee et al. (2021) [12]	RSF	Not Reported	0.9175	Not Reported	0.8963	Not Reported	0.8947	Not Reported	Not Reported
Longato et al. (2021) [13]	Deep Learning model with GRU	Not Reported	Not Reported	Not Reported	Not Reported	Not Reported	0.84	0.865	0.815
Kanda et al. (2022) [8]	XGBoost	Not Reported	Not Reported	Not Reported	Not Reported	Not Reported	0.752	Not Reported	Not Reported
Abegaz et al. (2023) [14]	XGBoost	0.8	0.94	0.76	0.84	0.89	0.8	0.83	0.79
	RF	0.81	0.91	0.78	0.82	0.86	0.8	0.82	0.78
	LR	0.66	0.63	0.67	0.89	0.74	0.66	0.68	0.64
	WEM	0.76	0.92	0.71	0.8	0.86	0.76	0.78	0.74
Gandin et al. (2023) [15]	PHNN	Not Reported	Not Reported	Not Reported	Not Reported	Not Reported	0.771	0.818	0.723
Wang et al. (2023) [16]	RF	Not Reported	Not Reported	Not Reported	Not Reported	Not Reported	0.978	Not Reported	Not Reported
	LGBM	Not Reported	Not Reported	Not Reported	Not Reported	Not Reported	0.873	Not Reported	Not Reported
	LR	Not Reported	Not Reported	Not Reported	Not Reported	Not Reported	0.87	Not Reported	Not Reported
	SVM	Not Reported	Not Reported	Not Reported	Not Reported	Not Reported	0.837	Not Reported	Not Reported
	CART	Not Reported	Not Reported	Not Reported	Not Reported	Not Reported	0.822	Not Reported	Not Reported
Alimova et al. (2023) [17]	NN	Not Reported	Not Reported	Not Reported	Not Reported	Not Reported	0.827	Not Reported	Not Reported
	LR	Not Reported	Not Reported	Not Reported	Not Reported	Not Reported	0.72	Not Reported	Not Reported
	GLM	Not Reported	Not Reported	Not Reported	Not Reported	Not Reported	0.702	Not Reported	Not Reported
	RF	Not Reported	Not Reported	Not Reported	Not Reported	Not Reported	0.768	Not Reported	Not Reported
	Extra Trees	Not Reported	Not Reported	Not Reported	Not Reported	Not Reported	0.726	Not Reported	Not Reported
Mora et al. (2023) [18]	Stacked Model	0.63	Not Reported	Not Reported	0.63	Not Reported	0.69	Not Reported	Not Reported
Nabrdalik et al. (2023) [19]	MLR	0.7245	0.7959	0.7083	0.3824	0.517	0.83	0.88	0.77
Sang et al. (2024) [20]	RF	0.664	0.664	0.664	Not Reported	Not Reported	0.722	0.783	0.66
	XGBoost	0.647	0.646	0.647	Not Reported	Not Reported	0.71	0.77	0.649
	LGBM	0.649	0.649	0.649	Not Reported	Not Reported	0.717	0.778	0.653
	ADB	0.653	0.653	0.653	Not Reported	Not Reported	0.716	0.779	0.654
	LR	0.654	0.654	0.654	Not Reported	Not Reported	0.717	0.791	0.636
	SVM	0.508	0.508	0.508	Not Reported	Not Reported	0.526	0.604	0.449
Soh et al. (2024) [21]	EBM	0.7938	0.89	0.62	0.82	0.853	0.81	0.794	0.787
Kwiendacz et al. (2025) [22]	LGBM	0.723	0.624	0.739	0.923	0.82	0.74	0.743	0.738
	RF	0.714	0.54	0.54	0.909	0.816	0.707	0.709	0.704
	SVC	0.686	0.698	0.614	0.918	0.792	0.707	0.71	0.704
	LR	0.59	0.596	0.589	0.899	0.711	0.71	0.713	0.707
	XGBoost	0.695	0.631	0.572	0.86	0.755	0.621	0.623	0.618
Bai et al. (2025) [5]	XGBoost	0.91	0.91	0.91	0.91	0.91	0.96	0.975	0.953
	RF	0.9	0.9	0.9	0.9	0.9	0.97	0.979	0.959
	LR	0.72	0.72	0.72	0.72	0.72	0.76	0.785	0.733
	SVM	0.81	0.81	0.81	0.81	0.8	0.86	0.883	0.841
	KNN	0.85	0.85	0.85	0.87	0.84	0.92	0.933	0.899
	LGBM	0.84	0.84	0.84	0.84	0.84	0.91	0.929	0.895
	ADB	0.76	0.76	0.76	0.76	0.76	0.83	0.855	0.811
	Neural Network	0.86	0.86	0.86	0.86	0.86	0.91	0.929	0.895
	Naive Bayes	0.69	0.69	0.69	0.69	0.69	0.73	0.759	0.705
Wändell et al. (2025) [23]	SGB	0.776	0.802	0.742	0.346	0.483	0.849	0.872	0.828

### 3.3 Heterogeneity Analysis

To examine the robustness of these pooled results, a leave‐one‐out sensitivity analysis was performed. The pooled sensitivity remained relatively stable, ranging between 0.80 and 0.86, regardless of which study was excluded. This indicates that no single study had an undue influence on the overall findings. However, heterogeneity assessments revealed that exclusion of the study by Sang et al. (2024) resulted in a substantial reduction in the Q statistic to 76.16, suggesting that this study contributed significantly to the observed heterogeneity. Conversely, removing the study by Wändell et al. (2025) slightly increased the between‐study variance, likely because the large sample size of this study helped to stabilise the overall meta‐analytic estimates.

In an effort to explore potential sources of heterogeneity, meta‐regression analyses were conducted using algorithm type and country of origin as moderators. The meta‐regression examining algorithm type did not yield statistically significant results (*p* = 0.080), although the model explained a large portion of the variance (*R* ^2^ = 0.96). Similarly, meta‐regression using country of study as a moderator showed no significant association with sensitivity (*p* = 0.074), with the model accounting for 87% of variance (*R* ^2^ = 0.87). These findings suggest that neither the type of machine learning algorithm nor the country in which the study was conducted sufficiently explained the heterogeneity observed among the included studies.

**FIGURE 4:**
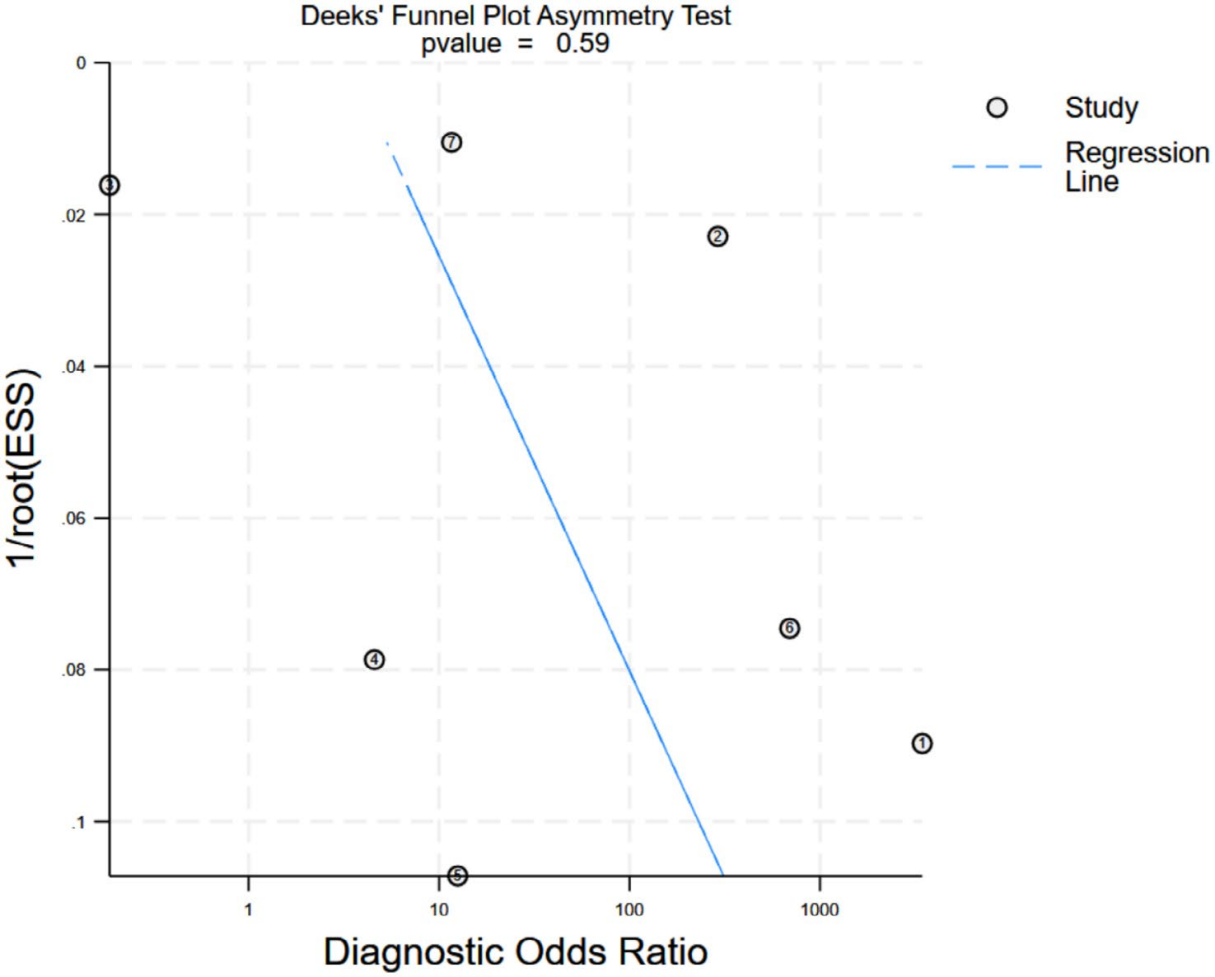
Funnel Plot for best performing models.

Publication bias was assessed using Egger's regression test applied to the sensitivity estimates. The test yielded a *p*‐value of 0.59, indicating no statistically significant evidence of publication bias (Figure 4). However, given the limited number of included studies, the power to detect bias was inherently low, and the possibility of unrecognised bias cannot be entirely excluded.

### 3.4 Quality Assessment

**FIGURE 5:**
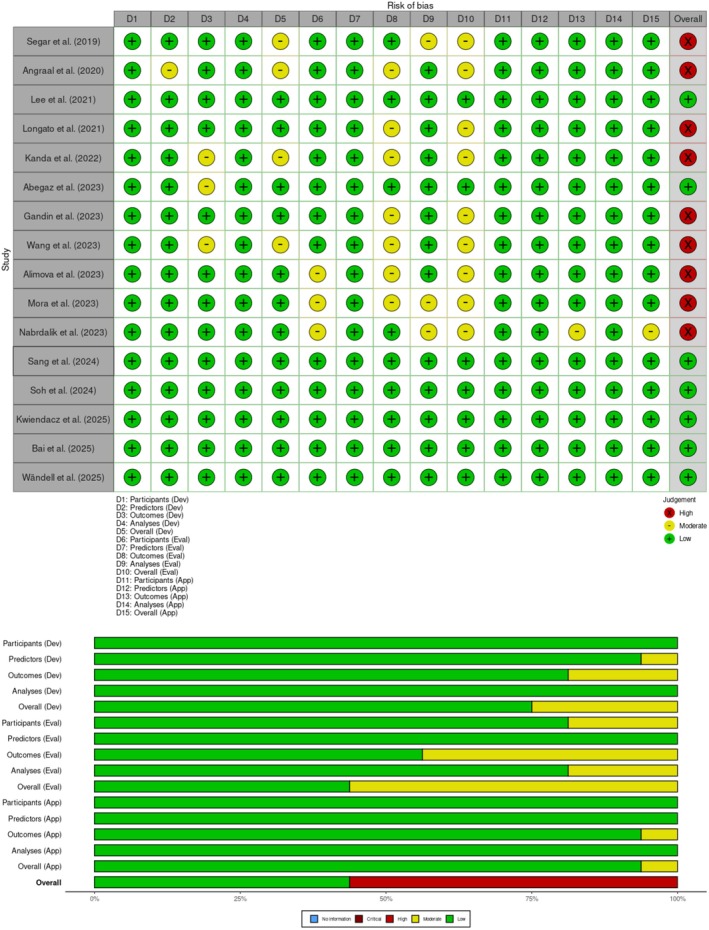
Quality assessment based on PROBAST + AI tool.

The quality of the included studies was evaluated using the PROBAST‐AI tool, focusing on three key domains: development, evaluation, and application. In the development domain, 75% of studies were assessed as having a low risk of bias, while 25% showed a moderate risk. The evaluation domain showed a greater proportion of concern, with 56% of studies rated as having a moderate risk of bias and 44% rated as low risk. The application domain reflected strong methodological adherence, with 94% of studies rated as low risk and only 6% as moderate risk. Overall, 56% of the studies were considered at high risk of bias when all domains were combined, while 44% were judged to be at low overall risk (Figure 5). These findings highlight methodological variability among the included studies, particularly in the evaluation phase, underscoring the need for more rigorous validation practices in future research.

### 3.5 GRADE Assessment

The certainty of evidence for the diagnostic performance of machine learning models assessed using the GRADE framework. The pooled sensitivity and specificity estimates were high; however, the overall certainty of evidence was rated as low due to several factors. The primary reason for downgrading was the high heterogeneity observed across studies, which raises concerns about the consistency and generalisability of the findings. Additionally, despite no significant publication bias detected by Egger's test, the small number of studies limits the confidence in this assessment, warranting a further downgrade for potential reporting bias. The indirectness of evidence was considered low since most studies directly addressed the clinical question. However, variability in study design, model types, and external validation approaches contributed to concerns about applicability. Imprecision was also noted, particularly for specificity and diagnostic odds ratio estimates, reflected by wide confidence intervals. Taken together, these factors led to an overall low certainty of evidence, suggesting that while the findings are promising, further high‐quality, standardised studies are needed to strengthen the confidence in the clinical applicability of ML models for heart failure detection.

## 4 Discussion

Analysis of the best‐performing ML models for heart failure detection showed a pooled sensitivity of 0.84 (95% CI: 0.75–0.90) and specificity of 0.86 (95% CI: 0.56–0.97), with an AUROC of 0.90 (95% CI: 0.87–0.93). These results suggest that the models can correctly identify 84% of patients with heart failure and accurately exclude 86% without it. The positive likelihood ratio of 6.6 (95% CI: 1.2–35.9) indicates a substantial increase in disease probability after a positive test, supporting their role in screening and diagnostic confirmation. Conversely, the negative likelihood ratio of 0.17 (95% CI: 0.08–0.36) means a negative result considerably lowers the likelihood of heart failure, aiding in its exclusion. The diagnostic odds ratio of 39 (95% CI: 4–423) underscores the strong overall discriminatory power of these ML models, highlighting their potential utility in clinical decision‐making for heart failure management.

The considerable heterogeneity observed among the included studies likely stems from differences in study populations, machine learning algorithms used, dataset sizes, and validation methods. Variability in patient populations—including age, comorbidities, disease prevalence, and diagnostic criteria—can influence both model performance and outcome measures, making direct comparison difficult. The choice of ML algorithms also contributes, as different models such as random forests, boosting methods, or support vector machines differ in learning approaches, risk of overfitting, and handling of data complexity, all of which affect diagnostic accuracy. Additionally, the size of datasets impacts both model training and evaluation, with smaller datasets increasing the risk of overfitting and producing less reliable results, while larger datasets generally enhance model stability and generalisability. Finally, the validation methods used across studies vary widely, ranging from internal validation to external testing on independent cohorts, directly influencing performance estimates. These methodological and clinical differences collectively contribute to the high heterogeneity observed and highlight the importance of standardised protocols, consistent validation strategies, and diverse patient inclusion in future ML research for heart failure detection.

Currently, despite the growing body of research on machine learning models for heart failure prediction, their translation into routine clinical practice remains limited. Most studies have focused on algorithm development and internal validation, with little emphasis on how these models could be embedded into everyday healthcare workflows. Segar et al. reported that a Random Survival Forest (RSF) model outperformed traditional methods, showing stronger predictive accuracy for heart failure with reduced ejection fraction (HFrEF) compared to heart failure with preserved ejection fraction (HFpEF), suggesting these models may have subtype‐specific utility [6]. In contrast, Kanda et al. found that the Gradient Boosting model (XGBoost) performed well on Japanese claims data, with AUROC values between 0.690 and 0.898, driven by variables like age and hospitalisation frequency, and maintained consistent performance in external validation [8]. Abegaz et al. demonstrated high sensitivity (0.94) for heart failure prediction using XGBoost on the All of Us dataset, particularly among type 2 diabetes patients on SGLT2 inhibitors, with key predictors including HbA1c and troponin [14]. Other studies reflected similar findings. Gandin et al. showed that a Physics‐Informed Neural Network (PHNN) performed well, achieving time‐dependent AUCs up to 0.780, especially when incorporating cardiac parameters and diuretic use [15]. Wang et al. reported an AUROC of 0.834 for a Random Forest model applied to NHANES data, with poverty‐income ratio and myocardial infarction as influential predictors [16]. Sang et al. validated a Random Forest model with an AUROC of 0.722, emphasising creatinine and HbA1c as significant features [20]. Kwiendacz et al. identified Light Gradient Boosting Machine (LGBM) as the top model (AUROC 0.740, sensitivity 0.62), particularly in chronic kidney disease patients with diabetes, driven by variables like eGFR and the TyG index [22]. Bai et al. reported high accuracy (0.91) and AUROC (0.96) for XGBoost in elderly diabetic patients, with the prognostic nutritional index (PNI) and fatty liver index (FLI) emerging as key factors [5].

### 4.1 Comparison With Traditional Risk Prediction Approaches

In contrast to the adaptability and predictive strength of machine learning models, traditional approaches rely on static biomarkers, fixed risk scores, or linear regression methods, making them less capable of capturing the complex and dynamic nature of heart failure risk. For example, applied clinical risk scores (such as WATCH‐DM) and biomarkers like NT‐proBNP and hs‐cTn in a cohort of over 6000 diabetic patients demonstrate that a two‐step screening strategy could reduce the number needed to screen and lower costs. However, this method relies on fixed thresholds and predefined variables, lacking the adaptability of machine learning models. In contrast, our reviewed ML models achieved stronger diagnostic shifts, as reflected by higher positive and negative likelihood ratios [24]. Echouffo‐Tcheugui et al. examined GDF‐15 over a 23‐year period and found a higher risk of heart failure in patients with elevated levels. Although the study showed robust longitudinal associations, its reliance on Cox regression limited its ability to account for complex interactions, such as sex‐specific effects, which machine learning models are better equipped to handle [25]. Berezin et al. assessed serum irisin for distinguishing heart failure phenotypes in diabetic patients, finding that while certain biomarkers improved phenotype‐specific predictions, the static nature of ROC‐based cutoffs limited broader applicability [26]. Said et al. developed a multivariable model in the ALTITUDE cohort that identified eight key predictors and achieved good discrimination (0.78 for sensitivity and 0.72 for specificity). However, even with added biomarkers, machine learning models have the capacity to incorporate a larger number of variables and can learn complex, non‐linear interactions among them. Furthermore, ML models can be retrained or fine‐tuned on different patient populations, allowing them to adapt to varying clinical contexts and demographic profiles [27]. These emphasise that while traditional approaches have contributed significantly to risk stratification, they often fall short in dynamic prediction and adaptability.

Together, these findings suggest that Random Forest and XGBoost are consistently strong performers, yet their success depends on the target population and chosen predictors. This variability reinforces the earlier point that, despite promising results, most models remain within academic or experimental settings. The inconsistency in performance and model sensitivity across studies highlights the need for context‐specific optimisation and better‐defined implementation pathways before these tools can be seamlessly adopted in clinical practice. Integration into electronic health records (EHRs) or clinical triage systems is seldom addressed in the literature, and few examples exist where ML tools have been successfully deployed at the point of care. As a result, the clinical utility of these models often stops at proof‐of‐concept or retrospective validation stages. Moreover, issues such as interoperability with EHR systems, seamless integration into clinical decision‐making processes, and usability by healthcare providers are rarely explored.

## 5 Study Limitations

This study has several limitations that should be considered when interpreting the findings. First, the number of included studies was relatively small, which may have limited the statistical power of subgroup analyses and meta‐regression, as well as the ability to detect publication bias. Second, substantial heterogeneity was present across studies, driven by differences in patient populations, machine learning algorithms, dataset sizes, and validation strategies, which may affect the consistency and generalisability of the pooled estimates. Third, we relied on the best‐performing model reported by each study without access to individual patient data, preventing a more detailed analysis of model development processes, calibration, and performance across different subgroups. Finally, most studies lacked external validation and used retrospective data, raising concerns about potential overfitting and real‐world applicability. These limitations highlight the need for cautious interpretation of the results and emphasise the importance of high‐quality, prospective studies with standardised methodologies in future research.

## 6 Conclusion

This meta‐analysis demonstrates that machine learning models achieve strong diagnostic performance for heart failure detection, with a pooled sensitivity of 84%, specificity of 86%, and an AUROC of 0.90 among the best‐performing models identified in each study. These findings underscore the potential of ML models to support early diagnosis and risk stratification, offering valuable assistance in clinical decision‐making. However, the presence of significant heterogeneity—largely due to differences in study populations, algorithms, dataset sizes, and validation methods—highlights the need for careful interpretation of pooled estimates and limits the immediate generalisability of these results. While the performance of ML models appears promising, especially in comparison with traditional risk prediction tools, their clinical impact remains dependent on addressing methodological and practical barriers.

Future research should aim to standardise machine learning development pipelines, including transparent reporting of model architecture, training processes, and evaluation criteria. Prospective validation studies across diverse patient populations and healthcare settings are essential to ensure external validity and to establish real‐world effectiveness. Efforts should also focus on improving model interpretability, enabling clinicians to understand and trust algorithmic decisions, which is critical for acceptance in routine care. Furthermore, the integration of ML models into clinical workflows must consider factors such as data quality, interoperability with electronic health records, and cost‐effectiveness analyses. Addressing these areas will be key to translating the promising diagnostic accuracy of ML models into meaningful improvements in patient outcomes and healthcare delivery.

## Author Contributions

Pooya Eini was a main contributor in the design, implementation, and writing of the manuscript. Mohammad Rezayee, Homa Serpoush, and Peyman Einiin dependently assessed articles and extracted data. All authors read and approved the final manuscript. Mohammad Rezayee performed statistical analysis.

## Ethics Statement

The authors have nothing to report.

## Consent

The authors have nothing to report.

## Conflicts of Interest

The authors declare no conflicts of interest.

## Supporting information


**Table S1:** Search syntax for different databases.

## Data Availability

Data sharing not applicable to this article as no datasets were generated or analysed during the current study.
