# Supplementary material for: Diagnostic Performance of Machine Learning Algorithms for Predicting Heart Failure in Diabetic Patients: A Systematic Review and Meta‐Analysis
Source: Endocrinol Diabetes Metab. 2025 Sep 18;8(5):e70111. doi: 10.1002/edm2.70111 (PMC12445121; doi:10.1002/edm2.70111)
Supplement: Supplementary file 1 — Table S1: Search syntax for different databases. [file EDM2-8-e70111-s001.docx]

| Supplementary table1. Search syntax for different databases | | |
| --- | --- | --- |
| database | syntax | results |
| pubmed | ("Heart decompensation"[Title/Abstract] OR "Heart Failure"[Title/Abstract] OR "Cardiac Failure"[Title/Abstract] OR "Myocardial Failure"[Title/Abstract] OR "heart failure" [MeSH Terms]) AND ("Machine learning"[Title/Abstract] OR "Artificial Intelligence"[Title/Abstract] OR "Machine Intelligence"[Title/Abstract] OR "Deep Learning"[Title/Abstract] OR "Computational Intelligence"[Title/Abstract] OR "Machine Learning"[Mesh]) AND ("Diabetes"[Title/Abstract] OR "Diabetes Mellitus"[Mesh]) | 226 |
| scopus | TITLE-ABS-KEY ( "Heart Failure" OR "Cardiac Failure" OR "Myocardial Failure" ) AND TITLE-ABS-KEY ( "Machine learning" OR "artificial intelligence" OR "Machine Intelligence" OR "deep learning" ) AND TITLE-ABS-KEY ( "Diabetes" ) | 983 |
| Web of science | (TS=("Heart Failure" OR "Cardiac Failure" OR "Myocardial Failure"))  AND (TS=("Machine learning" OR "Artificial Intelligence" OR "Machine Intelligence" OR "Deep Learning"))  AND (TS=("Diabetes")) | 655 |
| embase | 'machine learning'/de AND 'heart failure'/de AND 'diabetes mellitus'/de | 352 |
| proquest | TIAB("Heart Failure" OR "Cardiac Failure" OR "Myocardial Failure")  AND TIAB("Machine learning" OR "artificial intelligence" OR "Machine Intelligence" OR "deep learning")  AND TIAB("Diabetes") | 94 |
| EBSCO | ("Heart decompensation" OR "Heart Failure" OR "Cardiac Failure" OR "Myocardial Failure") AND ("Machine learning" OR "Artificial Intelligence" OR "Machine Intelligence" OR "Deep Learning" OR "Computational Intelligence") AND ("Diabetes") | 320 |
| Google scholar | ("Heart decompensation" OR "Heart Failure" OR "Cardiac Failure" OR "Myocardial Failure") AND ("Machine learning" OR "Artificial Intelligence" OR "Machine Intelligence" OR "Deep Learning" OR "Computational Intelligence") AND ("Diabetes") | 200 |
| total |  | 2830 |
